# Microenvironmental regulation of tumour immunity and response to immunotherapy

**DOI:** 10.1002/path.5681

**Published:** 2021-05-19

**Authors:** Mark M Kockx, Mark McCleland, Hartmut Koeppen

**Affiliations:** ^1^ CellCarta Antwerp Belgium; ^2^ Genentech South San Francisco CA USA

**Keywords:** immunotherapy, PD‐1, PD‐L1, tumour microenvironment, pembrolizumab, atezolizumab, nivolumab, ipilimumab, immuno‐oncology, TILs, immune oncology therapy resistance

## Abstract

The confluence of immunology and oncology has led to a lot of uncertainty and questions about relevant biomarkers. Despite the complexity of the tumour microenvironment, most clinical studies have relied on a single‐parameter immunohistochemical assay to prospectively select patients for checkpoint inhibitor therapy; the results of this strategy have been highly variable and often less than optimal. While great efforts have been made to identify additional or alternative biomarkers, pathologists, drug developers, and clinicians alike have faced technical, logistical, and regulatory challenges on how to implement them successfully. In this review, we will discuss these challenges; we will also highlight recent advances in dissecting the functional diversity of immune cell populations within the tumour microenvironment and their potential for improved, biomarker‐driven therapeutic strategies. The dynamic nature and cellular diversity of the tumour microenvironment may challenge past models of a single biomarker predicting patient response and clinical outcome. © 2021 The Authors. *The Journal of Pathology* published by John Wiley & Sons, Ltd. on behalf of The Pathological Society of Great Britain and Ireland.

## Introduction

The emergence of immunotherapy has truly revolutionised cancer treatment over the last decade. Numerous therapeutics directed against programmed cell death protein‐1 (PD‐1) or its ligand (PD‐L1) have been approved by regulatory agencies as monotherapy agents, in combination with chemotherapy, or in combination with other targeted agents. In clinical studies, PD‐L1 immunohistochemistry (IHC) and tumour mutation burden (TMB) have been the predominant predictive biomarkers explored to date; however, PD‐L1 IHC remains the dominant companion diagnostic test used in clinical practice to inform immune checkpoint inhibitor (CI) therapy.

PD‐L1 is expressed on the surface of tumour cells and tumour‐infiltrating immune cells. Many studies across a diversity of tumour types have established an association between PD‐L1 levels and clinical benefit to single‐agent anti‐PD‐1/PD‐L1 therapies [1, 2]. While patients with the highest level of PD‐L1 typically derive the greatest clinical benefit, the observation that patients whose tumours express low or no PD‐L1 still derive benefit has tempered our enthusiasm for this biomarker [3].

Considering the limitations of PD‐L1 as a single analyte biomarker, there is a high unmet need to understand the complex tumour microenvironment (TME) and develop better biomarkers and diagnostics that more accurately predict clinical benefit. It is crucial that we further explore the causal relationship between changes in the evolving cancer genome and infiltrating immune cell subtypes to generate an effective immune response against the cancer.

## Predictive biomarkers in immuno‐oncology drug development

### PD‐L1 IHC

The approval of PD‐L1 assays as companion diagnostic devices has cemented their use in defining standard of care therapies. The parallel approval of numerous PD‐L1 assays with different sensitivities, scoring algorithms, and approved cut‐off(s) has certainly generated a fair share of confusion among patients and health care providers. Moreover, the approval of these specific assays offers additional challenges for companies that continue to develop new drug combinations and/or new diagnostic tests [4]. As improved and more complex predictive biomarkers emerge, how do such assays replace (from a regulatory perspective) the current single‐plex assays?

Unlike the binary nature of driver mutations that report either mutant or wild‐type status, PD‐L1 is a linear biomarker and thus is amenable to evaluation at multiple expression thresholds. The approved PD‐L1 IHC assays employ not only different expression thresholds but also different scoring methods owing to the complex biology of PD‐L1 expression on both tumour and immune cells. PD‐L1 scoring algorithms are assay‐specific, indication‐dependent, and they change based on line of therapy. Certain assays score tumour cell expression only (and immune cells are ignored), while others exclusively evaluate PD‐L1 on immune cells (and tumour cells are ignored), while yet others measure PD‐L1 on both tumour and immune cells. The intricacies of PD‐L1 IHC assays have recently been reviewed in detail in refs 5, 6, 7. Table 1 illustrates the validated PD‐L1 IHC assay cut‐offs for the prominent PD‐L1 assays used in non‐small cell lung cancer (NSCLC). It should be noted that the approval and utility of these validated cut‐offs are country‐specific.

**Table 1:** Cut‐off values of PD‐L1 IHC assays associated with PD‐1 and PD‐L1 checkpoint agents for treatment of NSCLC.

	DAKO 28‐8	DAKO 22C3	VENTANA SP142	VENTANA SP263
PD‐L1 IHC assay cut‐off values	TC ≥ 1% TC ≥ 5% TC ≥ 10%	TPS ≥ 1% TPS ≥ 50%	TC ≥ 1% or IC ≥ 1% TC ≥ 5% or IC ≥ 5% TC ≥ 50% or IC ≥ 10%	TC ≥ 1% TC ≥ 5% TC ≥ 10% TC ≥ 25% TC ≥ 50%

IC, immune cell; TC, tumour cell; TPS, tumour proportion score.

**Figure 1:**
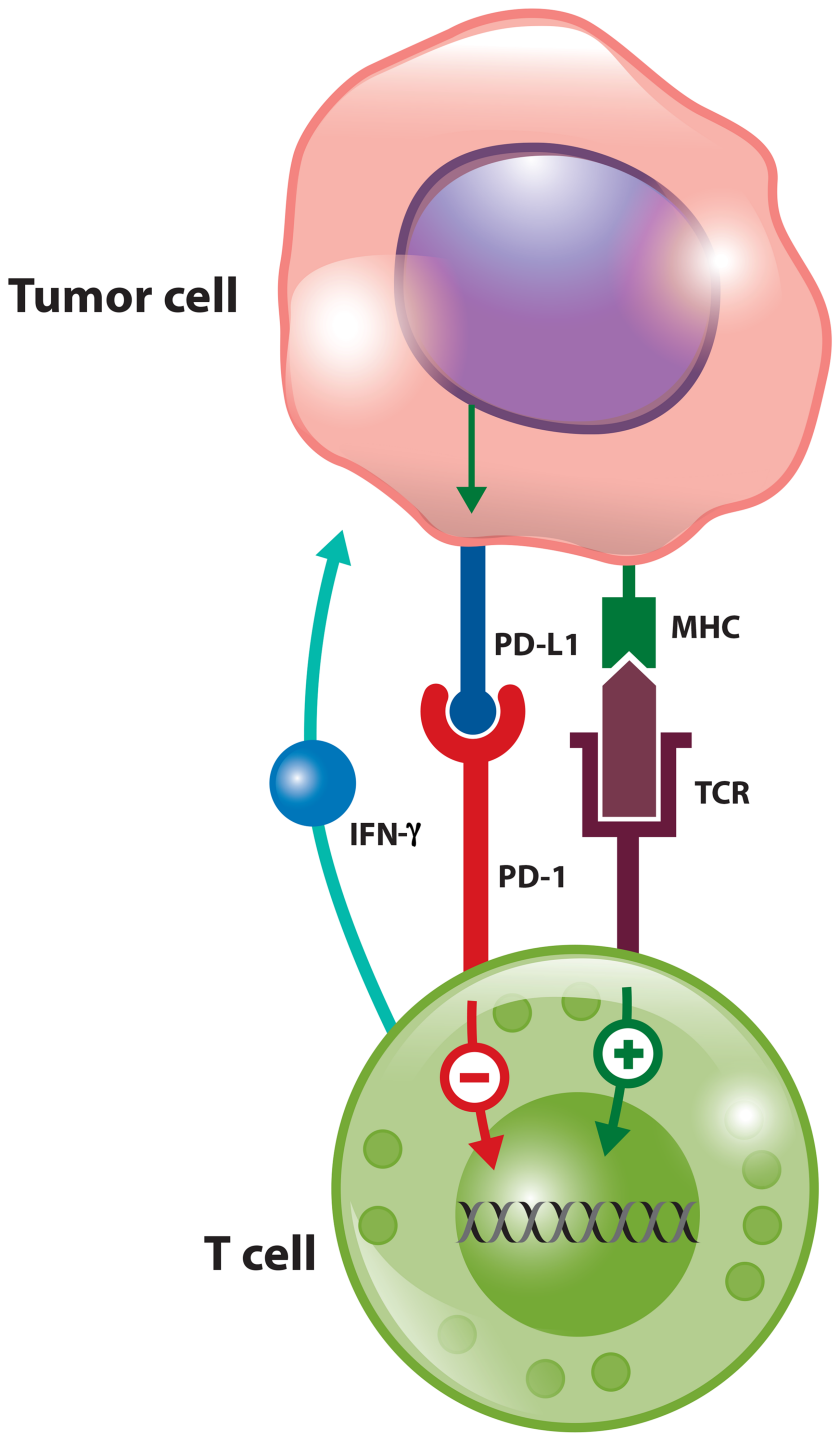
The relationship between T‐cell infiltration and PD‐L1 tumour cell expression.

The expression of PD‐L1 is dynamic and shows significant variation across tumour types, tumour sites, and even within the same tumour specimen [8, 9, 10]. Expression of PD‐L1 in tumour cells can be regulated by genomic, epigenetic, and transcriptional mechanisms [11]. The strong correlation between PD‐L1 on tumour cells and the extent of CD8^+^ T‐cell infiltration in NSCLC is most likely due to transcriptional regulation in response to inflammatory signals, such as IFN‐γ, that are produced by an active anti‐tumour immune response (Figure 1) [12, 13]. The non‐uniform expression of PD‐L1, commonly restricted to regions with immune infiltrates, suggests that PD‐L1 is adaptively induced as a consequence of high local concentrations of relevant cytokines within the TME and further highlights intra‐tumoural heterogeneity.

Intra‐tumoural heterogeneity and plasticity of PD‐L1 may affect patient management decisions for obvious reasons. A sample may yield a false‐negative PD‐L1 IHC result due to sampling artefact associated with small biopsies. Furthermore, location of a biopsy and prior therapy are important parameters for PD‐L1 expression. While obtaining a fresh biopsy at the time of treatment initiation may not be feasible for clinical reasons, an archival tumour specimen does not necessarily reflect the TME in recurrent or metastatic lesions, due to plasticity of the immune response in general and PD‐L1 in particular.

Multiple immune cell types, primarily macrophages, myeloid dendritic cells (DCs), but also T‐, NK‐ and B‐cells, can express PD‐L1 [14]. It is unclear whether specific immune populations or spatial features of immune cells are of most clinical relevance. Further characterisation of the spatial interplay of tumour cells with specific immune cell subsets using multiplex IHC technologies is needed to determine whether continued classification of immune cells as one broad category is warranted. More nuanced, complex assays are required [15].

### Beyond PD‐L1 IHC

Clinical trials have suggested that tumours with a high number of effector T‐cells are more responsive to CI therapy. Tumours with a low‐density immune infiltrate, or low numbers of T‐cells, are less responsive, highlighting the role of cytotoxic T‐cell biomarkers such as CD8 [16]. This in turn has stimulated the identification of gene expression signatures. Ayers *et al* used a gene expression signature centred on IFN‐γ to show associations with overall response rate (ORR) and progression‐free survival (PFS) for melanoma patients treated with the anti‐PD‐1 antibody pembrolizumab [17]. Most investigators relied on a combination of ‘IFN‐γ’, ‘T‐cell receptor (TCR) signalling’, and ‘expanded immune’ gene expression signatures. Consistent with these findings, the IMpower150 trial in NSCLC reported that a T‐effector (Teff) gene expression signature, composed of PD‐L1, CXCL9, and IFN‐γ, enriched for PFS benefit when comparing atezolizumab plus chemotherapy with chemotherapy [18]. Notably, the clinical benefit and proportion of patients captured by the Teff assay were similar to those reported for populations defined by an established PD‐L1 IHC assay (SP142).

Patients with tumours of high mutational burden have been reported to derive greater clinical benefit from CI therapy. Concordance studies of TMB and PD‐L1 IHC appear to suggest that these biomarkers are orthogonal and independently predict benefit from CI therapy. Consequently, pembrolizumab recently received regulatory approval in unresectable or metastatic TMB‐high solid tumours for patients who have progressed following prior treatment and who have no satisfactory alternative treatment options [19, 20].

As the search continues for a more predictive biomarker, it will be essential to move away from the single analyte approach and integrate the various biological processes that are responsible for carrying out an immune response. CIs simply release checks on T‐cells, which continue to rely on an intact antigen presentation system and sufficient neoantigen for priming and stimulation. While we have a long way to go, initial exploratory analysis has shown that combining biomarkers may translate into clinical benefit. In the Checkmate‐026 and Checkmate‐227 studies, patients whose tumours expressed high levels of PD‐L1 and had high tissue TMB status derived the greatest benefit from treatment with the anti‐PD‐1 antibody nivolumab, measured by ORR and PFS [21, 22, 23]. Similar results were reported in the OAK study, in which NSCLC patients with high PD‐L1 status and high blood TMB levels derived the greatest benefit when treated with the anti‐PD‐L1 antibody atezolizumab [24]. However, it is important to note that the association between high TMB and benefit from CI therapy is inconsistent across clinical studies. Similar to PD‐L1 IHC, different methodologies and assays have been employed to determine TMB and the read‐outs are poorly standardised, so our understanding of how TMB relates to expression of relevant neoantigens and a subsequent productive immune response remains incomplete [25, 26].

Owing to the heterogeneity of cancer and our current inability to fully model human disease in pre‐clinical models, the identification of precise predictive biomarkers often requires large, randomised clinical datasets. While retrospective analysis of phase 3 studies has shown promise beyond PD‐L1, we need to acknowledge that such data should be considered exploratory and hypothesis‐generating in nature only. Indeed, regulatory approval of a biomarker as a companion diagnostic test requires thorough analytical validation of the assay and prospective testing in a pivotal clinical study. How do we implement such biomarkers into clinical practice without re‐running phase 3 trials and obtaining a companion diagnostic label? How much retrospective data is sufficient to consider replacing PD‐L1 IHC without a new trial? Ideally, we will find ways to identify better biomarkers earlier in the clinical development of a molecule to prospectively implement in phase III studies. Even yet, a key challenge remains. PD‐L1 IHC and associated cut‐offs have regulatory approval and thus define a population and a specific treatment regimen (standard of care). In order for a new treatment regimen to obtain regulatory approval, it must demonstrate clinically meaningful and statistically significant improvement versus standard of care in that specific population, defined by a specific diagnostic assay. This leaves little room for the incorporation of new diagnostic assays, which ultimately may be more predictive, when trying to beat standard of care. As populations continue to be segmented based on the approval of targeted therapies, it presents additional complexities to drug development and the introduction of companion diagnostic tests for novel biomarkers.

## TME: phenotypic diversity of immune effector cells

Numerous studies have described the relationship between the presence and spatial distribution of tumour‐infiltrating immune cells and treatment benefit. The co‐localisation of CD8^+^ T‐cells and DCs has received particular attention. Johnson, Kerr, and colleagues, more than 20 years ago, observed that the distribution of T‐cells and dendritic cells in the TME of NSCLC was of prognostic significance [27]. In surgically resected specimens, the overall degree of immune cell infiltration was not prognostic. However, infiltration of CD3^+^ T‐cells and S100^+^ stellate cells, most likely DCs, within the tumour cell strands themselves correlated with improved survival, suggesting an ongoing anti‐tumour immune response. Although the detection of DCs is complex, the staining of S‐100^+^ stellate cells in the tumour strands, as observed in their study, is compatible with DC staining. As the overall levels of these cell types had no influence on survival, the authors stipulated the significance of this particular pattern to local chronic inflammation in tumours [27].

Guidelines to assess tumour‐infiltrating lymphocytes (TILs) in the stroma of breast cancer [28] and other tumour types [29] were published in an attempt to standardise prognostic findings and outline the clinical relevance to clinicians and their patients. These guidelines are detailed, and pathologists follow them in the context of interpretation of a haematoxylin and eosin (H&E)‐stained tumour section. Inter‐ and intra‐observer concordance of TIL assessment is variable but should benefit from implementation of appropriate training methodologies and resources [30, 31]. While traditional TIL assessment covers different types of immune cells recognisable in an H&E section, it considers TILs within the stromal compartment of a tumour lesion [28] and not intra‐epithelial immune cells. However, ‘inflamed’ tumours with intra‐epithelial immune cells represent a subgroup with distinct gene expression signatures, biological behaviour, and improved survival when compared with ‘excluded’ or ‘desert’ tumours [32, 33, 34]. We have focused on the detection of CD8^+^ T‐cells by IHC in our assessments of tumour immunophenotypes [35]. The capture of intra‐epithelial immune cells in routine H&E sections is unreliable [30], but the IHC signal lends itself for quantification by digital means and CD8 is a consistent component of gene expression signatures of ‘inflamed’ tumours [34].

Interestingly, the spatial distribution of stromal TILs in breast cancer has recently been re‐evaluated. The co‐localisation of CD11c^+^ DCs with areas of high‐density TILs in triple‐negative breast cancer (TNBC) may point to the presence of an ongoing immune response initiated and supported by DCs and other immune effector cells [36]. However, the effect on clinical outcome was not tested in this study. In inflammatory breast cancer (IBC), including TNBC, stromal TILs are associated with increased PD‐L1 and improved clinical outcome [37]; here, the co‐localisation of CD163^+^ myeloid cells (macrophages and/or DCs), not absolute numbers, was associated with achieving a pathological complete response [37].

Recognition of the spatial distribution of various immune effector cells needs to be complemented by functional analyses, such as activation marker and antigen reactivity, preferably at the single‐cell level [38]. Scheper *et al* analysed the intrinsic anti‐tumour reactivity of the intra‐tumoural TCR repertoire of CD8^+^ T‐cells in ovarian and colorectal cancer (CRC) samples and showed that the capacity to recognise autologous tumour is limited to a small minority of intra‐tumoural CD8^+^ T‐cells [39]. To make matters more complicated, significant TCR heterogeneity can exist within CD4^+^ and CD8^+^ T‐cell populations in different regions of the tumour (centre versus invasive margin), and the degree of heterogeneity carries prognostic significance. While remarkable, these observations were made on a rather limited NSCLC cohort, and confirmatory studies on independent samples are needed [40]. By extension, it appears likely that the TCR repertoire may differ in individual sites within a given patient. It also suggests that immune stimulatory approaches in conjunction with CI therapy may prompt exhausted or paralysed T‐cell populations to generate productive anti‐tumour immunity [41]. Heterogeneity of the intra‐tumoural T‐cell population is not limited to the TCR repertoire but extends to lineage‐determining and functional markers [42, 43]. Heterogeneity is observed among CD4^+^ and CD8^+^ T‐cells; however, more emphasis has been placed on CD8^+^ cells as they are believed to play the major role in the effector stage of tumour cell killing.

Typically, three distinct CD8^+^ T‐cell populations are identifiable based on common transcriptional profiles:

- naive or memory cells expressing CC‐chemokine receptor 7 (*CCR7*) and transcription factor 7 (*TCF7*);
- perforin 1 (*PRF1*), granzyme A (*GZMA*), and *GZMB*‐positive cytotoxic cells; and
- a diverse population of ‘dysfunctional’ cells characterised by markers typically associated with a state of exhaustion, such as PD‐1 (*PDCD1*), *LAG3*, and TIM3 (*HAVCR2*) [44, 45, 46].

The relative proportions of these three populations appear highly variable, not only across different tumour types but also across tumours of the same histology [44]. Overlay of transcriptional profiles and TCR sequencing information has been used to determine whether intra‐tumoural CD8^+^ cells phenotypically change and ‘differentiate’ from one pool to the other. A preliminary model suggests TME‐induced evolution of a phenotypically heterogeneous population of dysfunctional cells from naive CD8^+^ cells, whereas the latter group gives rise to cytotoxic cells in a TME‐independent manner. To what extent cells from the ‘dysfunctional’ pool can transition into a ‘cytotoxic’ state is currently unclear; however, this would be important to understand to design therapeutics that promote and trigger such transition [43].

Intra‐tumoural CD8^+^ cells with specificity for neoantigens expressed by the tumour have been identified in several studies but universally represent a small proportion of the total, phenotypically heterogeneous population of Teffs [47, 48, 49, 50]. Interestingly, CD39 appeared to distinguish tumour‐specific (CD39‐high) from bystander (CD39‐low) CD8^+^ cells. CD39^+^ cells also showed a less diverse TCR repertoire than CD39^−^ cells, suggesting clonal expansion of effector cells by stimulation with tumour‐associated neoantigens in small cohorts of NSCLC and CRC patients [50]. Additional phenotypic and functional analyses revealed that the CD39^+^ subset co‐expresses CD103 as well as PD‐1, TIM3, and CTLA‐4, markers typically associated with a state of exhaustion [51]. Co‐expression of CD39 and CD103 appeared dependent on prolonged stimulation through the TCR and exposure to TGF‐β. Interestingly, high levels of CD39^+^ CD103^+^ double‐positive cells appeared to translate into a survival benefit for patients with squamous cell head and neck cancers [51].

## TME: location matters

The phenotypic and functional diversity of intra‐tumoural CD8^+^ T‐cell populations has been established by several studies across multiple solid tumour types; this includes populations with variable proliferative and cytotoxic activity as discussed above. Generation and maintenance of a productive immune response requires interaction between effector cells and cells with antigen‐presenting and priming capabilities. Clinical response to CI therapy is thought to represent pre‐existent T‐cell‐mediated anti‐tumour immunity that can be unleashed following blockade of PD‐1/PD‐L1 interactions. On the other hand, lack of a clinical response may represent tumours in which the priming or promotion of a nascent immune response is defective; this was addressed in a recent paper analysing the spatial relationships between CD8^+^ T‐cells and MHC class II‐expressing cells, most likely antigen‐presenting cells (APCs) [52]. In a cohort of patients with renal cell carcinoma, TCF1^+^/CD8^+^ cells preferentially co‐localised to areas with clusters of MHC class II‐expressing cells, whereas TCF1^−^/CD8^+^ cells followed a more diffuse distribution throughout the tumour. The prevalence of TCF1^+^/CD8^+^ cells also correlated with that of DCs, whereas TCF1^−^/CD8^+^ cells did not show such correlation. Intriguingly, tumours with a high density of areas containing TCF1^+^/CD8^+^ T‐cells and MHC class II‐expressing APCs had a better clinical outcome compared with tumours with fewer clusters. The proposed model suggests that APC‐dense regions provide an intra‐tumoural milieu for stem‐like CD8^+^ T‐cells to differentiate into effector cells capable of sustaining an anti‐tumour immune response. The evidence for this model remains circumstantial; validation on larger cohorts and other tumour types is necessary. Furthermore, understanding the mechanisms underlying the generation of APC clusters – and possibly their destruction in CI‐resistant tumours – would be crucial in designing therapeutic interventions that foster anti‐tumour immunity.

It has become evident that tumour infiltration of mature, active DCs into the tumour bed confers an increase in immune activation and recruitment of active immune effector cells. Accurate identification and quantification of DCs would be of value; however, markers such as CD11c, CD11b, CD163, and MHC‐II are not DC‐specific and are expressed on other cell types such as macrophages. The observation that DCs can promote or suppress a nascent immune response further adds to the complexity, as there are currently no validated markers available that discriminate between both DC states [53].

**Figure 2:**
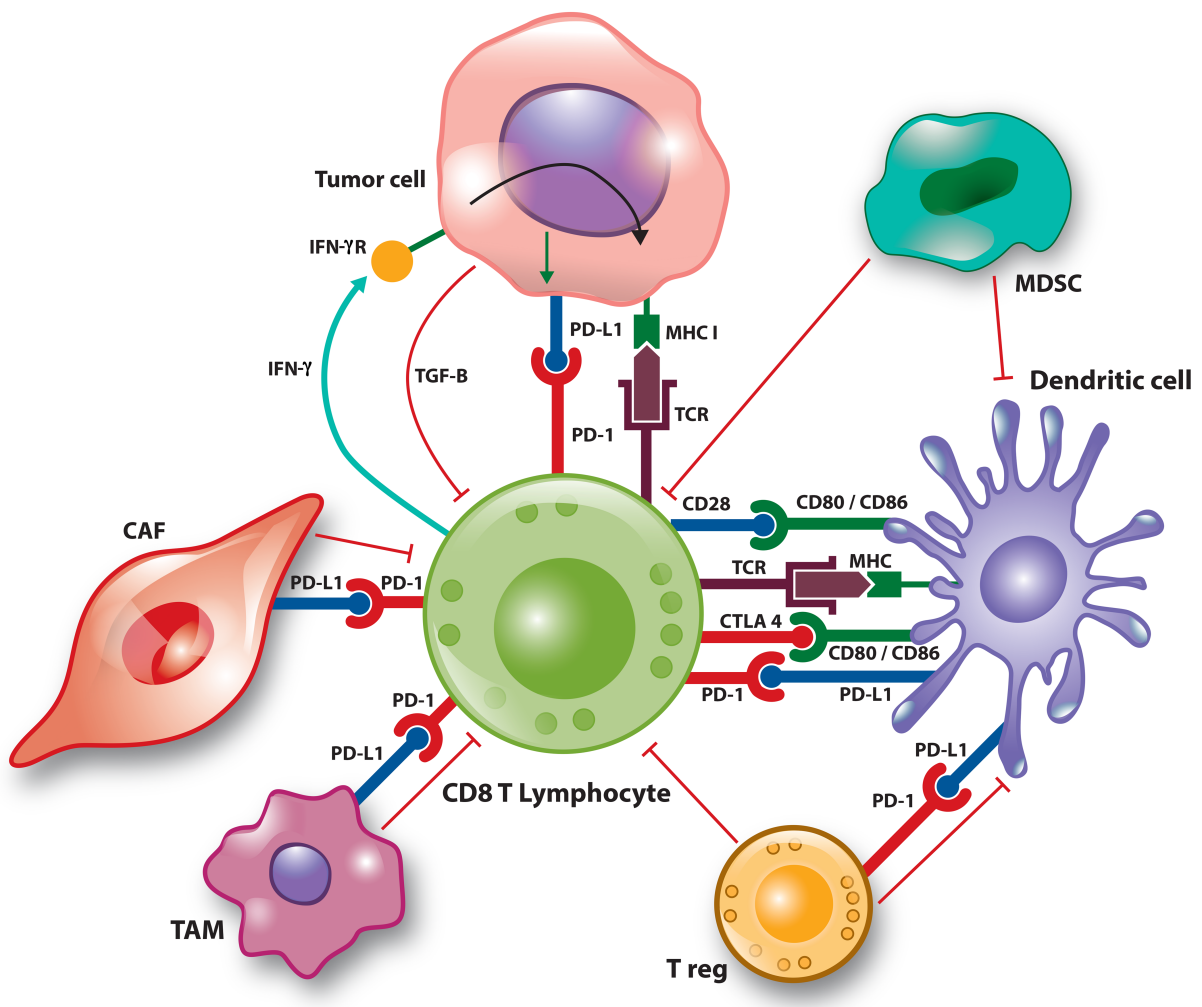
Tumour protector cells of the TME are T regulatory cells (Tregs), tumour‐associated macrophages (TAMs), cancer‐associated fibroblasts (CAFs), and myeloid‐derived suppressor cells (MDSCs).

During tumour progression, the TME changes into an environment of active protection of tumour cells from immune attack and, by extension, from immunotherapy. Cells of the TME that have been ascribed tumour‐protective function are T regulatory cells (Tregs), tumour‐associated macrophages (TAMs) [54], cancer‐associated fibroblasts (CAFs) [55, 56, 57], and myeloid‐derived suppressor cells (MDSCs) (Figure 2). Tumour‐protective effects are typically mediated through secreted cytokines and chemokines, which prevent immune effector T‐cells from invading into the tumour bed and/or may render such cells functionally ineffective [58, 59]. For example, TGF‐β secreted by CAFs has been proposed to lead to the exclusion of T‐cells from the TME in patients with advanced bladder cancer [35]. Response to PD‐L1‐targeted therapy was associated with the presence of CD8^+^ Teffs, whereas tumour progression was seen in tumours with an expression signature dominated by TGF‐β signalling in fibroblasts. The latter observation correlated with tumours showing a CD8^+^ T‐cell infiltrate primarily in the fibroblast‐ and collagen‐rich peritumoural stroma. Similar observations were published concurrently for a mouse model of microsatellite‐stable CRC (MSS‐CRC). In this model, mice with genetic defects in pathways commonly mutated in human CRC [60, 61, 62] copy the clinical features of advanced human CRC, facilitating analysis of pathogenetic mechanisms as well as evaluation of novel therapeutics. Tauriello *et al* demonstrated that mutant mice develop locally advanced and metastatic tumours with an immune‐excluded phenotype, accumulation of PD‐1^+^ T‐cells, PD‐L1^+^ stromal cells, and evidence of active TGF‐β signalling [63]. Combined treatment with a TGB‐β inhibitor and anti‐PD‐L1 antibody caused intra‐tumoural influx of Teffs, changing the phenotype from ‘excluded’ to ‘inflamed’ and reducing the number of metastases and improving long‐term survival [63]. It will be interesting to see if these promising pre‐clinical observations can be duplicated in patients with MSS‐CRC, a tumour type that has proven resistant to most forms of CI therapy [64, 65, 66].

MDSCs are a heterogeneous population of neutrophil‐ and monocyte‐like myeloid cells that are increasingly recognised as key mediators of immunosuppression in various types of cancer [67, 68]. In cancer patients, increased numbers of circulating MDSCs correlate with advanced clinical stage, increased incidence of metastatic disease, and immunosuppression. MDSCs can mediate immunosuppression through multiple mechanisms including the production of reactive oxygen species and depletion of key amino acids required for T‐cell proliferation through expression of arginase and indoleamine 2,3‐dioxygenase [59, 69]. In addition, MDSCs produce a range of immunosuppressive and cancer‐promoting cytokines, including interleukin‐10 (IL‐10) and TGF‐β. Besides their immunosuppressive function, MDSCs may also actively shape the tumour microenvironment through complex crosstalk with tumour cells and surrounding stroma, resulting in increased angiogenesis, tumour invasion, and metastasis.

## Intrinsic resistance of tumour cells and the TME

Understanding the differential response and resistance mechanisms to cancer immunotherapy is a critical gap in the field. While we hope to find a silver bullet, the likely truth is that there are many mechanisms that contribute to resistance. Tumours are constantly evolving and selecting ways to evade an immune system response, even in the absence of an administered drug. While increasing mutational load through alterations in tumour suppressors like p53 should amplify the number of neoantigens and sensitise tumours to the immune system, these mutations can also drive mutations in antigen‐presenting pathways and/or in key cellular machineries that can trigger transcriptional and metabolic changes that in turn have indirect effects on tumour‐infiltrating immune cells.

A key component of the tumour antigen presentation machinery is MHC class I (MHC‐I), which is composed of an HLA class I (HLA‐I) subunit and β2‐microglobulin (β2M). Downregulation of MHC‐I has been reported in a broad range of tumour types and can occur through several mechanisms including genetic mutation, epigenetic silencing, transcriptional changes, and post‐translational processes [70, 71, 72]. Mutations in β2M and HLA‐I have been described across indications that lead to irreversible loss of MHC‐I. Mutations in upstream MHC‐I regulators, such as JAK1/2, seem to downregulate IFN‐γ signalling and MHC‐I expression. Interestingly, the proprotein convertase subtilisin/kexin type 9 (PCSK9), independent of its role in cholesterol metabolism, can function to downregulate MHC‐I by disrupting MHC‐I recycling to the cell surface [73]. Inhibition of PCSK9 with function‐blocking antibodies sensitised pre‐clinical mouse tumour models to CI. It will be of value to identify additional therapeutic targets that can enhance MHC‐I expression to increase benefit from existing CIs.

How does downregulation of MHC‐I impact the TME? Perea *et al* analysed the density and composition of tumour T‐cell infiltration in NSCLC in relation to PD‐L1 and HLA‐I expression [74]. Positive HLA‐I expression correlated with the density of the immune infiltrate, independent of PD‐L1 status. All HLA‐I‐positive/PD‐L1‐positive tumours had a high degree of CD8^+^ T‐cell infiltration, whereas HLA‐I loss was associated with a reduced number of intra‐tumoural T‐lymphocytes, which were spatially limited to the stroma surrounding tumour nests. HLA‐I‐negative/PD‐L1‐positive tumours were larger and showed a lower density of CD8^+^T‐cells. This study suggests a cancer immune escape phenotype that combines two independent mechanisms of immune evasion: loss of HLA‐I and upregulation of PD‐L1.

Molecular analyses of tumours that are void of a productive CD8^+^ T‐cell infiltrate suggest that activation of oncogenic pathways in tumour cells can impair induction or execution of a local anti‐tumour immune response. Analysis of metastatic melanomas revealed that more than 30% of the lesions were non‐inflamed, and of those non‐inflamed lesions, 48% showed evidence of activation of WNT–β‐catenin signalling within tumour cells [75].

T‐cell‐inflamed and non‐inflamed tumour lesions can be present within the same patient or even within the same tumour lesion, suggesting that in some cases, features within specific metastatic sites may prevent T‐cell‐mediated immunity [76]. Alterations in the cytokine milieu of the TME may determine the presence or absence of productive anti‐tumour immunity in individual metastatic sites without genetic changes in antigen processing or presentation pathways. We need to understand the interaction between intra‐tumoural heterogeneity driven by intrinsic tumour alterations and immune cell infiltration and makeup within the TME. The presence of an immune infiltrate is associated with selective pressure on tumour cells to find ways of immune escape. Mechanisms of escape vary not only between tumours but also within an individual tumour lesion and are dictated by density and functional characteristics of a local immune population, as recently shown in an elegant study of NSCLC [77].

## Lessons learned from the neoadjuvant setting

The recent exploration of immunotherapy in early‐stage disease has provided new opportunities to understand the TME and how it changes in response to therapy. Neoadjuvant studies are particularly informative because a diagnostic biopsy is frequently collected prior to treatment and can be compared to the surgical resection taken immediately after the neoadjuvant therapy. Retrospective analyses on three neoadjuvant bladder cancer trials, ABACUS, PURE‐01, and NABUCCO, have yielded new insights [78, 79]. The ABACUS trial was a single‐arm phase 2 study investigating two cycles of neoadjuvant atezolizumab prior to cystectomy. Elevated levels of PD‐L1 (SP142; IC ≥ 5%), Teff gene expression, and intra‐epithelial CD8^+^ cells in baseline samples correlated with clinical benefit. Of note, baseline TMB and genes in the DNA damage response (DDR) pathway did not correlate with clinical outcome and thus differ from findings in the metastatic setting. Interestingly, increased numbers of intra‐epithelial CD8^+^ cells and PD‐L1^+^ immune cells were observed in the post‐therapy tumour sample compared with pre‐surgery, and this increase was more pronounced in responders than in patients who relapsed. Elevated TGF‐β, fibroblast activation protein, and cell cycle genes were found to be associated with resistance in post‐treatment samples.

Similar results were reported in the PURE‐01 study, where urothelial bladder patients received three cycles of pembrolizumab before radical cystectomy. Patients with elevated PD‐L1 IHC [22C3; combined positive score (CPS) ≥ 10%] achieved a higher pathological complete response rate than those patients with low PD‐L1 status. Of interest, median PD‐L1 CPS levels increased and median TMB levels decreased in post‐therapy resections compared with pre‐therapy biopsies. Moreover, immune‐related genes, including those related to IFN‐γ signalling, antigen presentation, and T‐cell function, also increased in post‐treatment samples compared with pre‐treatment samples.

The NABUCCO study evaluated pre‐operative treatment with two doses of the anti‐CTLA4 antibody ipilimumab and two doses of nivolumab prior to resection in stage III urothelial cancer [80]. In contrast to the ABACUS and PURE‐01 studies, treatment outcome was not associated with the baseline status of intra‐tumoural CD8^+^ infiltrate or Teff signatures. Instead, the authors observed a trend between higher TMB and a higher frequency of DDR gene alterations in patients achieving a complete response. Patients unable to achieve a complete response were enriched in a TGF‐β gene expression signature in the baseline sample, suggesting a possible resistance mechanism. Van Dijk also examined the correlation between density of TILs and response; interestingly, there was no correlation between baseline TILs and response, but an enrichment in TILs was observed in post‐therapy tumours for patients achieving a complete response.

These observations are not unique to bladder cancer. The NEOSTAR trial evaluated nivolumab or nivolumab plus ipilimumab as neoadjuvant treatment in patients with operable NSCLC. Patients achieving radiographic or major pathological response had overall higher tumour cell PD‐L1 from baseline tumour samples. Immune profiling of post‐therapy resection by flow cytometry discovered a higher frequency of TILs, tissue‐resident memory T‐cells, Teffs, and effector memory T‐cells following nivolumab plus ipilimumab treatment compared with nivolumab therapy alone. Multiplex IHC analysis on pre‐ and post‐treatment samples also revealed an increase of TILs in tumours treated with nivolumab plus ipilimumab but not in tumours treated with nivolumab [81].

These findings warrant validation in larger randomised datasets to confirm predictive and resistance markers in the neoadjuvant setting and to understand more deeply how the early‐stage TME differs from metastatic disease. While there are some inconsistencies between biomarker status and benefit in these neoadjuvant trials, there are trends emerging. In general, baseline PD‐L1 appears to predict response to CI, and CI appears to drive immune cells into the tumour. Biomarkers such as TMB, TILs, and DDR alterations appear promising but require further evaluation and validation. Differences in treatment regimens, as well as heterogeneity in patient populations, may contribute to discordant findings in different trials. As neoadjuvant immunotherapy and chemo‐immunotherapy combination regimens gain regulatory approval, identifying validated biomarkers to predict immune response will be critical to help inform treatment decisions.

## Concluding thoughts

As the transition from chemotherapy to targeted and immune therapies continues to develop, investigators will utilise more complex clinical trial designs, such as combination of targeted and immune therapies, combination of different CI drugs, and designs that focus primarily on biomarkers with greater sensitivity to predict response or resistance to single or multi‐agent therapy regimens. It is quite likely that multi‐parametric approaches are needed to identify patients most likely to respond to CI treatment. Pathologists are poised to characterise the TME and to analyse the interaction between tumour and effector immune cells and between effector cells and APCs. The expression profile of relevant markers may suggest activation or suppression/exhaustion of effector cells or may hint at mechanisms of resistance. It will be important to develop robust tissue‐based multiplex technologies that can be adopted for routine clinical practice and – if necessary – successfully shepherded through regulatory approval.

## Author contributions statement

MMK, MM and HK contributed to writing and editing the manuscript and agreed to the final version.

**Abbreviations**
APC: antigen‐presenting cell
CAF: cancer‐associated fibroblast
CI: immune checkpoint inhibitor
CPS: combined positive score
CRC: colorectal cancer
DC: dendritic cell
DDR: DNA damage response
IBC: inflammatory breast cancer
IHC: immunohistochemistry
MDSC: myeloid‐derived suppressor cell
MHC‐I: MHC class I
MSS‐CRC: microsatellite‐stable CRC
ORR: overall response rate
PD‐1: programmed cell death protein‐1
PD‐L1: programmed cell death protein ligand 1
PFS: progression‐free survival
TAM: tumour‐associated macrophage
Teff: effector T‐cell
TIL: tumour‐infiltrating lymphocyte
TMB: tumour mutation burden
TME: tumour microenvironment
TNBC: triple‐negative breast cancer
